# Supplementary figures and images for: Inhibition of a transcriptional repressor rescues hearing in a splicing factor–deficient mouse
Source: Life Sci Alliance. 2020 Oct 21;3(12):e202000841. doi: 10.26508/lsa.202000841 (PMC7652395; doi:10.26508/lsa.202000841)

# Fig 2B

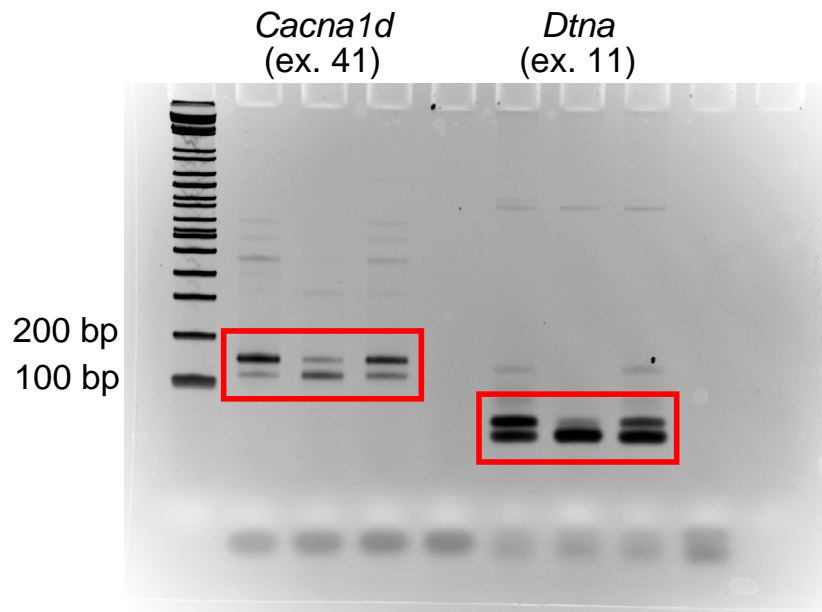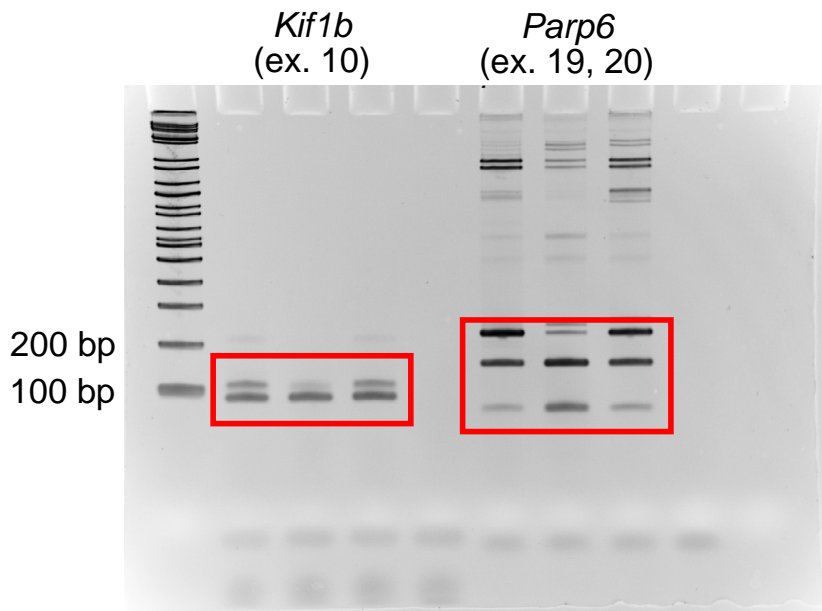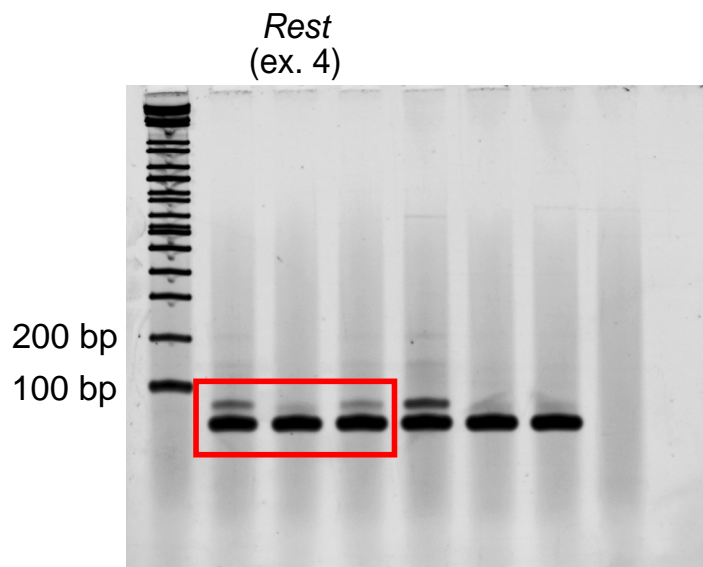

Supplement: Supplementary file 1 [file LSA-2020-00841_SdataF2.pdf]

# Fig 3G

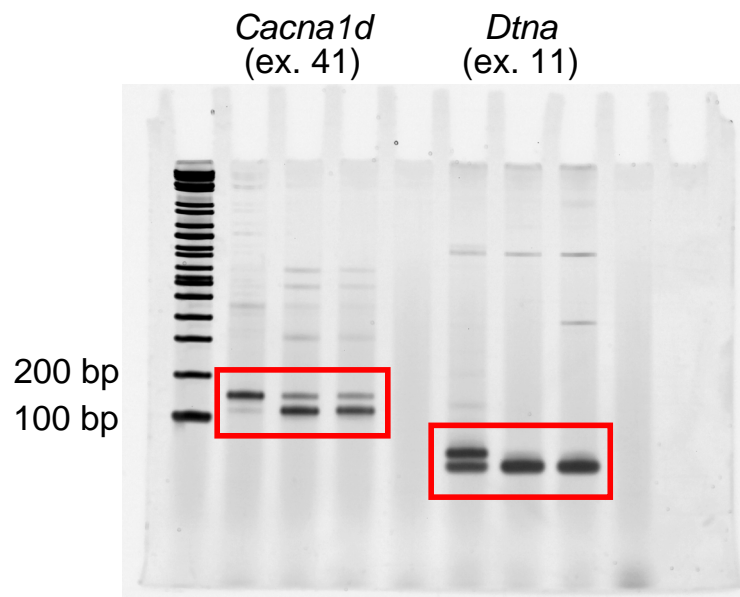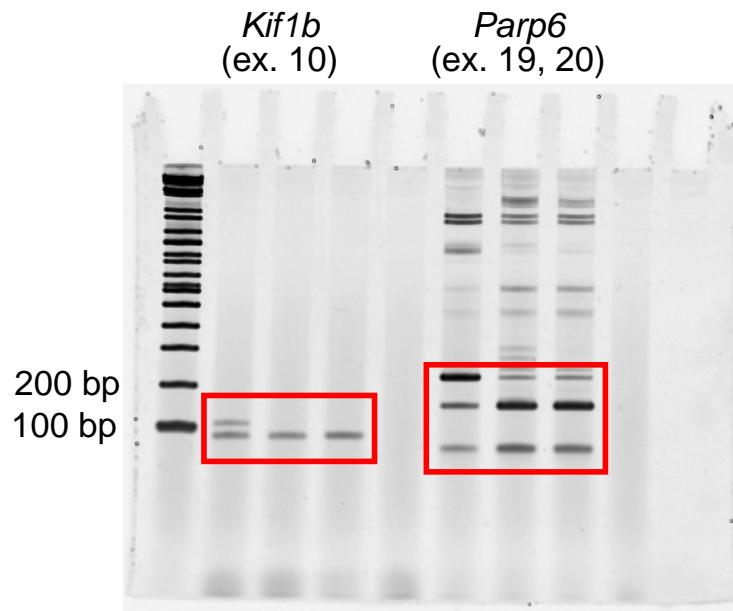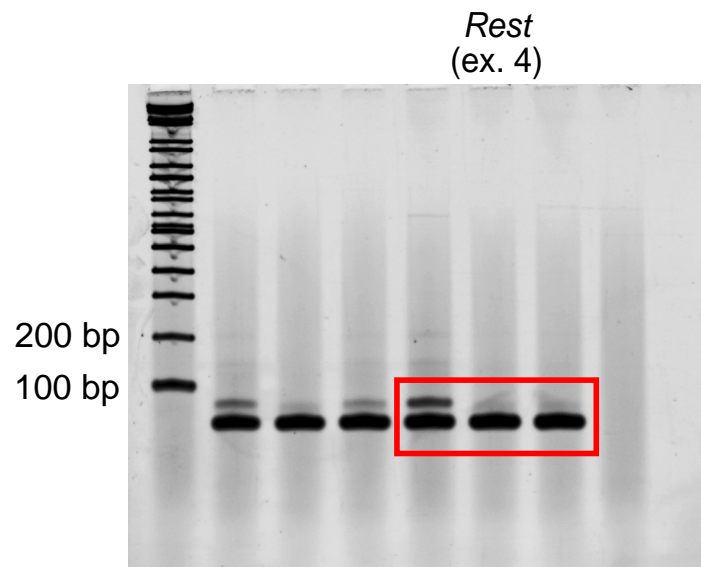

Supplement: Supplementary file 2 [file LSA-2020-00841_SdataF3.pdf]

# Fig 4D

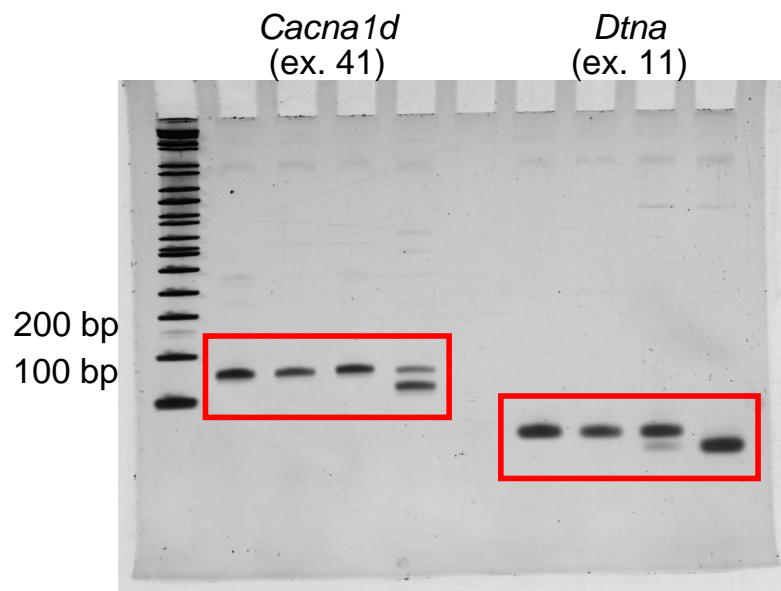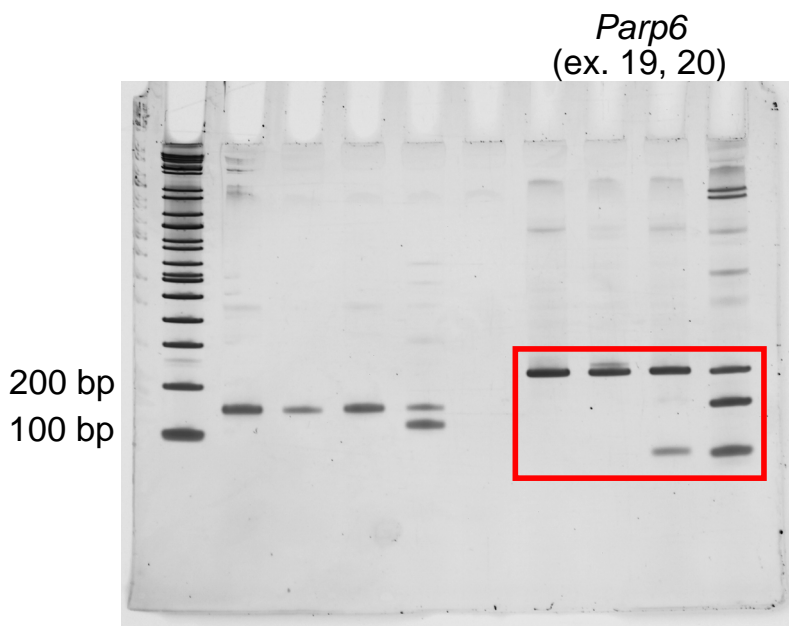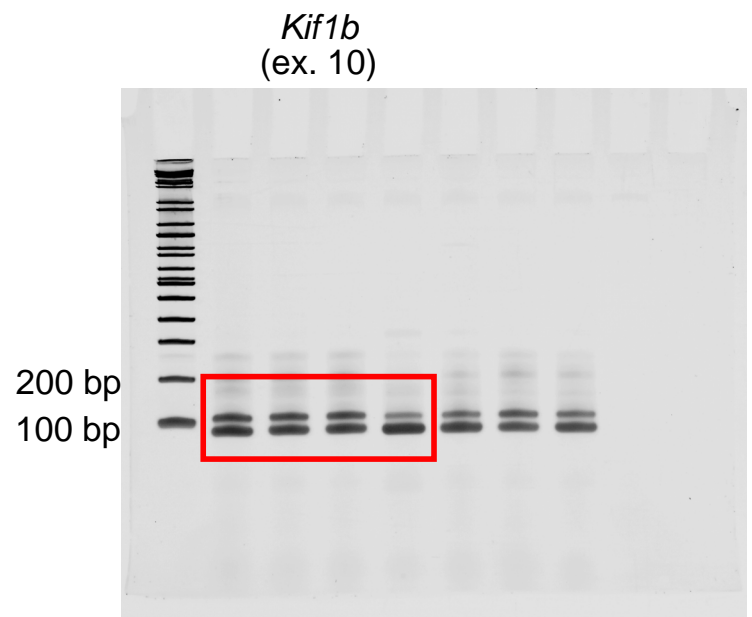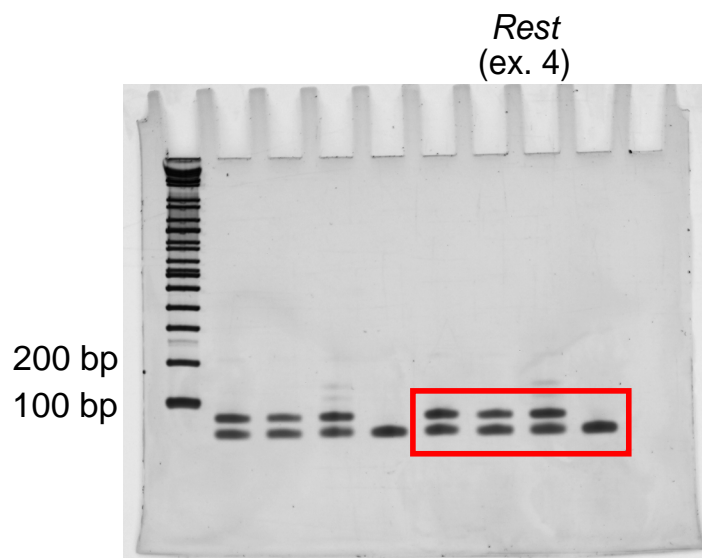

Supplement: Supplementary file 3 [file LSA-2020-00841_SdataF4.pdf]
